# Hospital Facts and Observations

**Published:** 1830-10-01

**Authors:** 


**Hospital Facts and Observations, illustrative of the Efficacy of the New Remedies, Strychnia, &c. &c. &c. By *Jas. Lomax Bardsley*, M.D. &c. 8vo, pp. 223. London, 1830.**

We have already noticed Dr. Bardsley's observations on strychnia and iodine, and we shall now devote a short article to the properties of the acetate of morphia, veratria, and colchicum. Our author has found the morphia beneficial in some cases of pain at the pit of the stomach.

*Case* 1. A young woman had laboured, for several months, under severe pain at the epigastrium, for which she had used leeches, blisters, and opium without succcess. The latter remedy afforded temporary relief, but produced excessive costiveness. A fourth of a grain of the acetate of morphia was given every two hours, and this not producing much effect, the quantity was ultimately increased to a grain every third hour, the bowels being kept open by mild aperients. She recovered perfectly in the course of a few weeks.

*Case* 2. Ellen Broton, fustian cutter, æt. 41, had laboured under severe pain at the scrobiculus cordis for more than six months; her appetite was nearly gone, and her bowels alternately loose and costive. Castor oil and a quarter of a grain of the acetate every two hours were prescribed, and followed by almost immediate relief. The dose was diminished, and the patient, in four or five weeks, dismissed cured.

*Case* 3. Michael Sharples, æt. 29, an industrious man, with a numerous family, had experienced much pain at the stomach occasionally for several months, and so severe had it been for the last three weeks, that he could not follow his usual employment. His spirits were low, appetite gone, and bowels costive. Leeches and a blister had been tried. The bowels being opened by purging medicine, a quarter of a grain of the acetate was given three times daily, with excellent effect. In five weeks he was discharged cured, but he subsequently applied with a relapse, which was combated successfully by the same plan of treatment.

*Case* 4. A young man had suffered, for some months, from such a gnawing pain at the stomach, that he could not continue his occupation, was reduced to poverty, and at last attempted to poison himself by laudanum. After a short course of aperients, Dr. Bardsley gave half a grain of the acetate twice daily; so much relief ensued that the dose was diminished; and, after the expiration of several weeks, he perfectly recovered both his mental and bodily strength.

We need not multiply these cases, but we ought to mention that our author has tried the salt for that troublesome symptom of dyspepsia, pyrosis. He only details one case.

*Case* 5. Mary Ford, a weaver, æt. 26, had always been delicate, but suffered severely, for the last two months, from gnawing pain at the pit of the stomach, copious discharge of an acid fluid from the mouth, flatulence, and capricious appetite. After castor oil, the acetate was prescribed, a fourth of a grain thrice daily, and in six weeks the patient was discharged cured.

Dr. Bardsley alludes to two or three cases of dyspepsia, in which the morphia was productive of little or no relief, and then passes on to some cases of uterine disease, in which he tested its palliative and remedial powers.

CASE. 1.—*Scirrhus Uteri.* A woman, æt. 46, had suffered for some months from severe lancinating pains in the hypogastrium, accompanied with a copious sanious, and very offensive discharge from the vagina. Her sufferings were heart-rending, her strength had much declined, and the inguinal glands were indurated and enlarged. Opium to some extent had been tried, but, although it afforded relief, it produced constipation, and then became a source of aggravation to her sufferings. An eighth of a grain of acetate of morphia was given every second hour, and shortly increased to half a grain every hour. Her sufferings were mitigated, her bowels no longer confined, and the path to the grave was smoothed. On dissection, the uterus was in a completely scirrhous state.

CASE 2. *Induration of the Uterus.* A woman, ætatis 36, was the subject of frequent shooting pains in the hypogastrium, costive bowels, and debility. On introducing the finger into the vagina, the uterus was felt to be harder than natural, somewhat enlarged, and very tender to the touch. Calomel, which brought away indurated fæces, then gentle laxatives, and the acetate of morphia were prescribed, and although the bowels continued to be confined, her appetite and strength improved, the uneasiness subsided, and our author discontinued his attendance.

CASE 3. *Painful Menstruation.* A female servant, ætatis 30, was subject to the most excruciating agony at each return of the menstrual discharge, indeed so great was the suffering that she generally remained insensible for some hours. Her general health appeared to be good, and her bowels were regular; she had tried various remedies without relief. She was ordered to take a quarter of a grain of the acetate of morphia on the first accession of pain, and to continue this proportion every half hour whilst it was violent. The second dose afforded her very great relief, without in the least suppressing the discharge, and she now takes the morphia at every monthly period, with the same advantage.

We are next presented with some cases of neuralgia in which the morphia was used with advantage or success.

*Case* 1. Ann Jones, ætatis 45, unmarried, was seized in April, 1826, with acute pain, shooting from the fore-part of the left cheek to the ala of the nose and angle of the mouth. After that she regularly experienced a recurrence of the pain, at first once in each week, afterwards several times daily, with the effect of impairing her general health. Every means almost imaginable had been tried, but ether in large doses mostly procured a temporary mitigation of her agony. The acetate of morphia was given, and on the third day its beneficial effects were apparent. At the expiration of three weeks the disease had completely yielded, and the patient has remained well ever since.

*Case* 2. Mary Williamson, ætatis 52, was first attacked in March, 1825, with severe lancinating pain in the site of the right mental foramen, which afterwards appeared at various times, and was the cause of two teeth being extracted, without relief. In June she came under our author's care, and then the least degree of motion or attempt at mastication produced a renewal of the pain. The acetate of morphia, with attention to the bowels, was employed, and in July the patient was discharged cured.

*Case* 3. John Hall, æt. 36, had laboured under inferior dental neuralgia for more than two years when he came under Dr. Bardsley's care. The nerve had been divided with temporary benefit, and opium taken largely with relief, although it constipated greatly. Morphia was exhibited, and in five weeks the patient was dismissed cured. In the course of a few months he reapplied with a relapse, and again the morphia answered for a while. The disease has returned, and he is now taking the extract of belladonna with advantage.

*Case* 4. Ralph Lomas, a weaver, had been subject for sixteen months to agonizing pain in the left cheek and outside of the nose. Latterly the paroxysms appeared several times in the day, and his sufferings were most distressing. The stomach and bowels were much disordered, and his constitution was impaired. Having paid attention to the chylopoietic viscera, Dr. B. tried the acetate, and the patient soon derived benefit from its use. In three months he was discharged cured.

" The above examples seem sufficient to establish the remedial efficacy of the acetate of morphia in several affections. In proof of its successful administration, it would not be difficult to adduce more instances; but it is my wish to avoid any further detail of cases than may be considered requisite to prove the value of the remedy in question. It must be allowed, that morphia has not always answered the intentions with which it has been employed, but the proportion of the favourable cases has been considerable, compared to those in which it has failed. I have never witnessed any *pernicious consequences* from a prudent use of the morphia. I am led to recommend the acetate of morphia in preference to opium, from a conviction that its efficacy may be equally relied upon, whilst its administration will be unattended by the *distressing head-ache, excessive constipation, and other unpleasant symptoms*, which that drug in large doses mostly induces. It appears to be the *chief advantage* of morphia, that it may be employed in those cases in which it is desirable to obtain a narcotic effect, and at the same time of the first importance to avoid constipation. It is proper to unload the bowels before commencing with the acetate, so as to give the remedy a fair chance. It is prudent to commence with not more than a quarter of a grain, which may be gradually increased to half a grain, a grain, or two grains, according to the urgency of the symptoms and the effect produced. These doses are applicable to adults. I have mostly prescribed it in the form of pill, which seems to answer very well.

It is desirable that the profession should put the virtues of the acetate of morphia to the test of experience, in order that its claims to notice may be better and more fully determined. It is only by the united observations of many practitioners that the value of any remedy can be satisfactorily established." 108.

So much for the acetate of morphia, and it only remains for us to advert to the properties of veratria and colchicum, as tested by Dr. Bardsley. The former substance is obtained from the veratrum *sabadilla*, and a few grains of the acetate have been found to destroy animals when introduced into the stomach and intestinal canal. Feeling anxious to ascertain its effects upon the human body, Dr. Bardsley exhibited it in several dropsical cases. The following may be a specimen of all.

*Case.* Hugh Cairns, æt. 39, had been ill three months with ascites, and anasarca of the lower extremities, scanty urine, feeble pulse, and somewhat difficult respiration. He commenced with a quarter of a grain of veratria every four hours, which not acting on the bowels with energy, was augmented to half a grain at the same intervals. It produced several watery motions daily, the swellings diminished, the dose was again augmented to a grain twice daily, and the patient was in the course of a month discharged cured.

We see no great prospect of veratria being likely to supersede our other hydropic medicines. Our author has made experiments on the colchicum autumnale in gout and rheumatism, and furnishes a table of twenty-four cases of that obstinate disease which were treated by veratria, and the same number put under the influence of colchicum. There does not appear to be much discrepancy in the results, but we need not pursue this inquiry farther. Every practitioner in these isles must have satisfied himself fully of the powers of colchicum, and the too frequently intractable nature of chronic rheumatism under any plan of treatment.

We have given the preceding cases and remarks without comment of our own, because they do not seem to require any. We fully agree with Dr. Bardsley that the properties of a drug are only ascertained by the united experience of many and various practitioners, and if our notice of these cases shall attract the attention of our brethren to the acetate of morphia we shall feel amply satisfied.

